# Liprin-α-1 is a novel component of the murine neuromuscular junction and is involved in the organization of the postsynaptic machinery

**DOI:** 10.1038/s41598-017-09590-7

**Published:** 2017-08-22

**Authors:** Krzysztof M. Bernadzki, Marta Gawor, Marcin Pęziński, Paula Mazurek, Paweł Niewiadomski, Maria J. Rędowicz, Tomasz J. Prószyński

**Affiliations:** 1 Laboratory of Synaptogenesis, Polish Academy of Sciences, 3 Pasteura Street, Warsaw, 02-093 Poland; 2 Laboratory of Molecular Basis of Cell Motility, Nencki Institute of Experimental Biology, Polish Academy of Sciences, 3 Pasteura Street, Warsaw, 02-093 Poland

## Abstract

Neuromuscular junctions (NMJs) are specialized synapses that connect motor neurons to skeletal muscle fibers and orchestrate proper signal transmission from the nervous system to muscles. The efficient formation and maintenance of the postsynaptic machinery that contains acetylcholine receptors (AChR) are indispensable for proper NMJ function. Abnormalities in the organization of synaptic components often cause severe neuromuscular disorders, such as muscular dystrophy. The dystrophin-associated glycoprotein complex (DGC) was shown to play an important role in NMJ development. We recently identified liprin-α-1 as a novel binding partner for one of the cytoplasmic DGC components, α-dystrobrevin-1. In the present study, we performed a detailed analysis of localization and function of liprin-α-1 at the murine NMJ. We showed that liprin-α-1 localizes to both pre- and postsynaptic compartments at the NMJ, and its synaptic enrichment depends on the presence of the nerve. Using cultured muscle cells, we found that liprin-α-1 plays an important role in AChR clustering and the organization of cortical microtubules. Our studies provide novel insights into the function of liprin-α-1 at vertebrate neuromuscular synapses.

## Introduction

The function of neuromuscular connections depends on the ability of muscle cells to maintain a high density of postsynaptic components, including acetylcholine receptors (AChR), at the cell surface^1–3^. Abnormalities in the clustering, stabilization, and turnover of AChR are often associated with severe neuromuscular disorders, such as myasthenia gravis^4^. There are an estimated 300 diseases of the neuromuscular system, and approximately 50% of them have an unknown etiology. The dystrophin-associated glycoprotein complex (DGC) is a multiprotein transmembrane complex. In muscles, it serves as a major laminin receptor that plays an important role in muscle integrity^5, 6^. At the neuromuscular junction (NMJ), the DGC is involved in stabilizing postsynaptic machinery by linking it to the extracellular matrix and actin-cytoskeleton^5, 6^. Mutations in several components of the DGC are known to cause muscular dystrophies that are often fatal^4, 7^. Apart from its function in skeletal muscles, the DGC was also shown to regulate many important processes, including cell migration, tumor progression, blood-brain barrier formation, and synaptic plasticity in the central nervous system (CNS)^6, 8–10^. Although the molecular mechanism that underlies the DGC-mediated stabilization of postsynaptic components is not fully understood, the cytoplasmic protein α-dystrobrevin-1 (αDB1) has been shown to play an important role in this process. αDB1 binds to dystrophin, utrophin, and syntrophin and regulates the dynamics of AChR at the NMJ^11–14^. To gain further insights into the molecular function of αDB1, we recently performed a biochemical screen for αDB1-interacting partners and identified liprin-α-1^12^ as one of these.

Liprin-α-1 belongs to the liprin family of cytosolic scaffold proteins, which consists of four liprin-α and two liprin-β isoforms in mammals^15^. α-Liprins form homodimers and heterodimers with β-liprins^15, 16^. Lyssand *et al*. reported that liprin-α-1 in HEK-293 cells interacts with α-catulin, another component of the DGC that also plays a crucial role in the organization of AChR clustering^12, 17^. Liprin-α-1 was additionally shown to interact with the LL5β-ELKS-CLASP complex that forms a specialized plasma membrane platform that is involved in regulating focal adhesion turnover, cortical microtubules, cell migration, and tumor cell invasion^18–21^. LL5β, ELKS, and CLASP are also associated with the local exocytosis and organization of intracellular vesicle transport^22, 23^. LL5β and CLASP were shown to be involved in the organization of AChR clusters in cultured myotubes, AChR exocytosis, and microtubule attachment at the postsynaptic specialisation^24–28^.

Different liprin-α isoforms are differentially expressed in various brain regions, suggesting their specialized functions. In CNS neurons, liprin-α proteins were found at both presynaptic and postsynaptic compartments where they act as a molecular anchor for receptors and other proteins during synaptic assembly^29–31^. The function of liprins in the nervous system was also studied in invertebrates. A mutation of liprin-α homolog that is present in *Drosophila* (dliprin-α) displayed impairments in the accumulation of synaptic vesicles along the axon, in addition to fewer boutons and a reduction of the synaptic complexity of larval NMJs^32–34^. Similarly, a mutation of the *C*. *elegans* homolog of liprin-α, SYD-2, was associated with diffused localization of synaptic vesicles in motor neurons and defects in the assembly of active zones. Consequently, SYD-2 mutant worms are characterized by uncoordinated movements and defects in egg-laying behaviour as a result of impairments in synaptic transmission^35–39^.

Despite the well-documented function of liprin-α in the nervous system, the localization and function of this protein in vertebrate NMJs has not been reported. In the present study, we characterized the localization of liprin-α-1 at the murine NMJ and found evidence that this protein is implicated in the organization of postsynaptic machinery.

## Results

### Liprin-α-1 is localized to contractile machinery and concentrates at the NMJ

**Figure 1:**
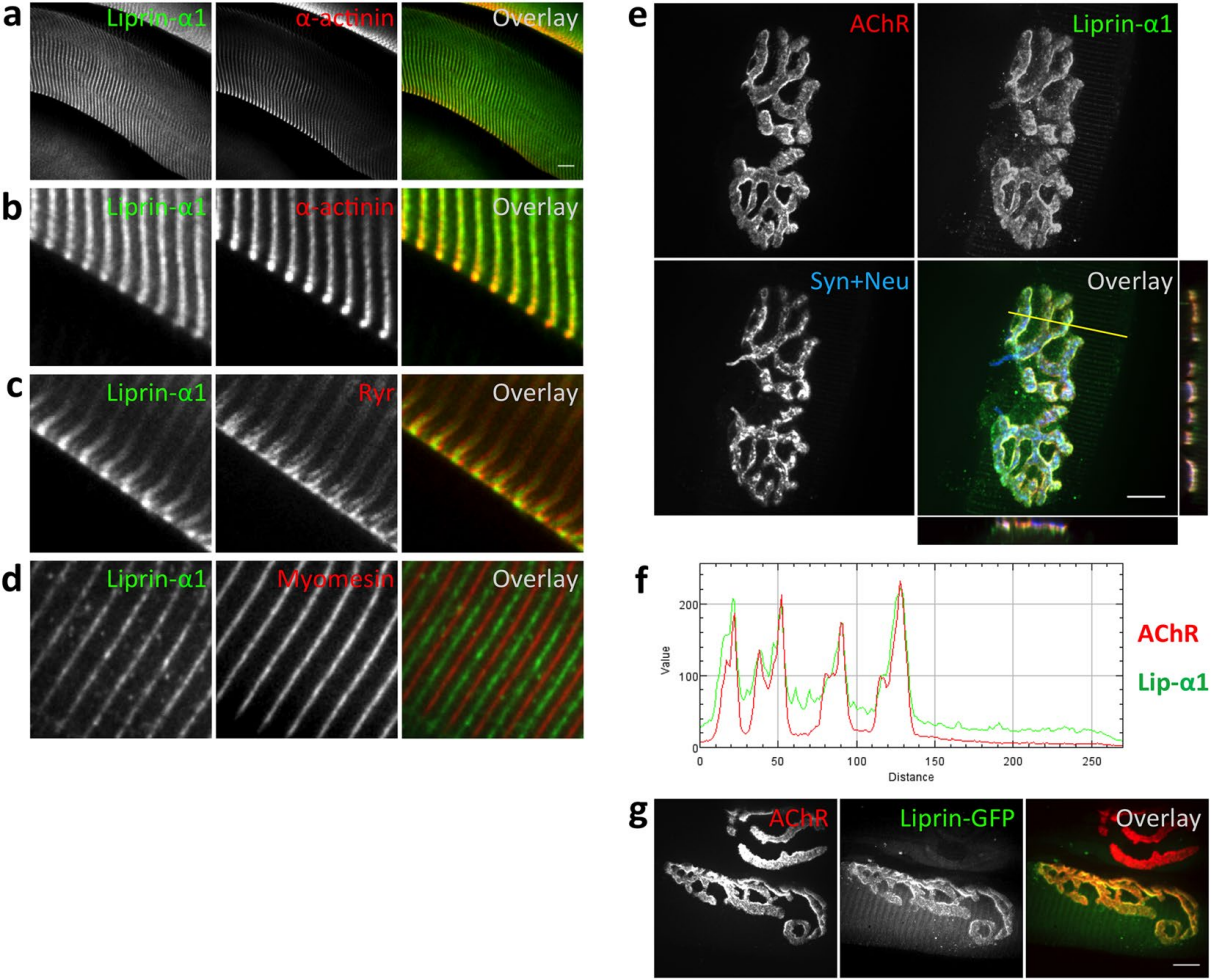
Liprin-α-1 localizes to Z-discs and NMJs in skeletal muscles. (**a**–**d**) Liprin-α-1 localized to the Z-discs of contractile machinery. Tibialis anterior muscle fibers from P30 mice were stained with antibody against liprin-α-1 protein (green) and indicated markers for contractile machinery (red). (**b**) Shows a higher magnification of an area in (**a**). (**e**) Liprin-α-1 (green) was enriched mainly at the postsynaptic machinery of the NMJ. Fluorescently labeled BTX was used to visualize AChR (red), and anti-synaptophysin and anti-neurofilament were used to visualize nerve terminals (blue). (**f**) Enrichment of liprin-α-1 at the postsynaptic machinery. Fluorescence intensity for liprin-α-1 immunochemistry (green) and AChR (BTX, red) along the line shown in (**e**) was plotted using RGB profiler plugin to the ImageJ/Fiji software. (**g**) Liprin-α-1-GFP fusion protein localized to the NMJ on transfected fiber. AChR (red); liprin-α-1-GFP (green). Scale bar 10 μm.

Previous studies highlighted a critical role for the DGC in muscle fiber integrity and in the development of the postsynaptic machinery^6^. Using a biochemical screen, we recently identified liprin-α-1 as a binding partner for αDB1, a cytoplasmic component of the DGC^12^. Liprin-α-1 has not been reported at the vertebrate NMJ. Therefore, we performed a histochemical analysis of its localization in mouse skeletal muscles. Liprin-α-1 immunoreactivity was detected in a striated pattern along the muscle fiber, which could be attributable to its association with a specific domain of contractile machinery. We performed co-localization experiments using various markers of contractile machinery, including α-actinin (an actin-binding protein that anchors myofibrillar actin thin filaments and titin to Z-discs; Fig. 1a,b), ryanodine receptor Ca^2+^ channels (RyRs) that are located in sarcoplasmic reticulum abutting Z-discs (Fig. 1c), and myomesin (a myosin crosslinking protein that is mainly present at the M-line; Fig. 1d)^40–42^. Immunohistochemical analyses of liprin-α-1 on fibers that were isolated from tibialis anterior muscles showed that it is predominantly associated with Z-discs that are enriched in α-actinin (Fig. 1a,b).

Strong liprin-α-1 immunoreactivity was also detected at neuromuscular synapses, visualized by fluorescently-labelled bungarotoxin (BTX) that specifically binds to the postsynaptic AChR (Fig. 1e,f). Optical cross-sections of confocal Z-stack images showed that liprin-α-1 was predominantly concentrated at the postsynaptic machinery (below or at the same level as AChR) but was also present at the presynaptic compartment where it co-localized with synaptophysin and neurofilament (Fig. 1e). We confirmed this observation in an independent experiment in which we ectopically expressed liprin-α-1-green fluorescent protein (GFP) fusion protein in muscle fibers and confirmed that it localized to the postsynaptic machinery (Fig. 1g). Similarly, overexpressed liprin-α-1-GFP was enriched at the AChR clusters in myotubes derived from C2C12 myoblasts or primary myoblasts (Supplementary Fig. 2a,b).

### Liprin-α-1 NMJ localization during development and in various types of muscles

**Figure 2:**
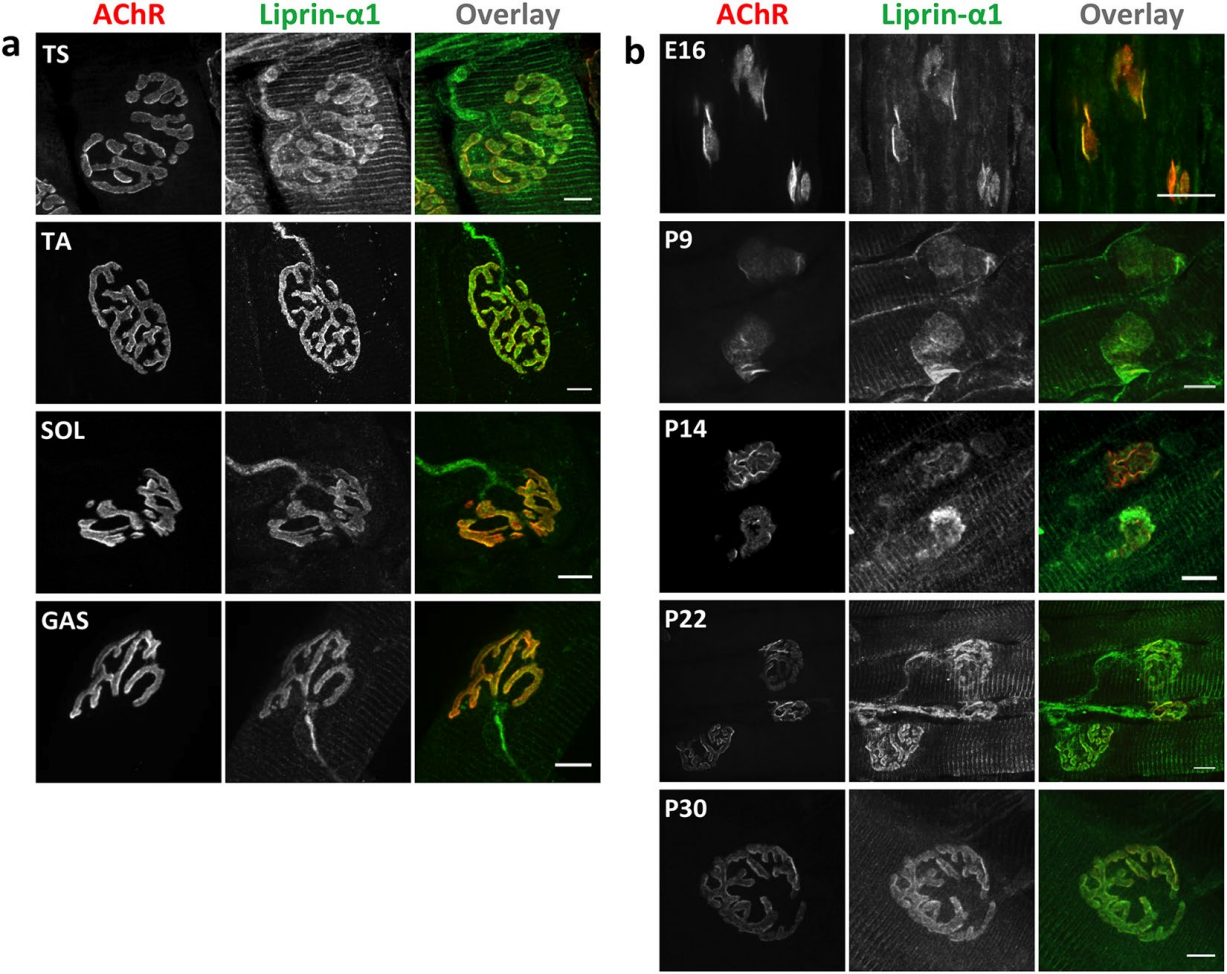
Localization of liprin-α-1 is similar in various muscles and maintained throughout the development. (**a**) Liprin-α-1 immunoreactivity in different muscles. TS, triangularis sterni; TA, tibialis anterior; SOL, soleus; GAS, gastrocnemius. Muscles were collected on P30. (**b**) Immunohistochemical analysis of TA muscle fibers on the indicated embryonic (E) and postnatal (P) days. Anti-liprin-α-1 antibody (green); AChR (red). Scale bar 10 μm.

Fibers in different muscles vary in their molecular composition and physiological properties^43, 44^. They can be divided into groups of either fast or slow contraction, based on the types of myosin heavy chain isoforms that are expressed. Tibialis anterior, triangularis sterni, and gastrocnemius muscles comprise predominantly fast fibers, and soleus muscles consist of predominantly slow fibers^43, 44^. Muscle fibers in different groups of muscles can also vary in their synaptic organization, reflected by the overall size and shape of NMJs^45^. Importantly, Chakkalakal *et al*. also found molecular differences between NMJs on fast and slow fibers^46^. Thus, liprin-α-1 expression and localization in various muscle types need to be investigated. We found that anti-liprin-α-1 antibody immunoreactivity was similar in tibialis anterior, triangularis sterni, gastrocnemius, and soleus muscles and presented a striated pattern along the fibers and strong enrichment at the NMJ (Fig. 2a).

During the early postnatal period, NMJs undergo intensive remodelling. Until postnatal day 7 (P7), clusters of postsynaptic machinery mostly grew in size, and the transformation of juvenile oval synapses into topologically complex structures occurred between P7 and P30 (Fig. 2b). Specific synaptic components could be recruited to the synapse at particular time points of development and maturation^12, 26, 47^. To study the presence of liprin-α-1 at the NMJ during these transitional stages, we performed immunohistochemical analyses of tibialis anterior muscles from mice at embryonic day 16 (E16) and at different postnatal days: P9, P14, P22, and P30. We observed a stable and constant level of liprin-α-1 during all stages of development. Liprin-α-1 was present at juvenile NMJs before their perforation, and similar localization was apparent at later stages (Fig. 2b).

### Localization of liprin-α-1 is nerve-dependent

**Figure 3:**
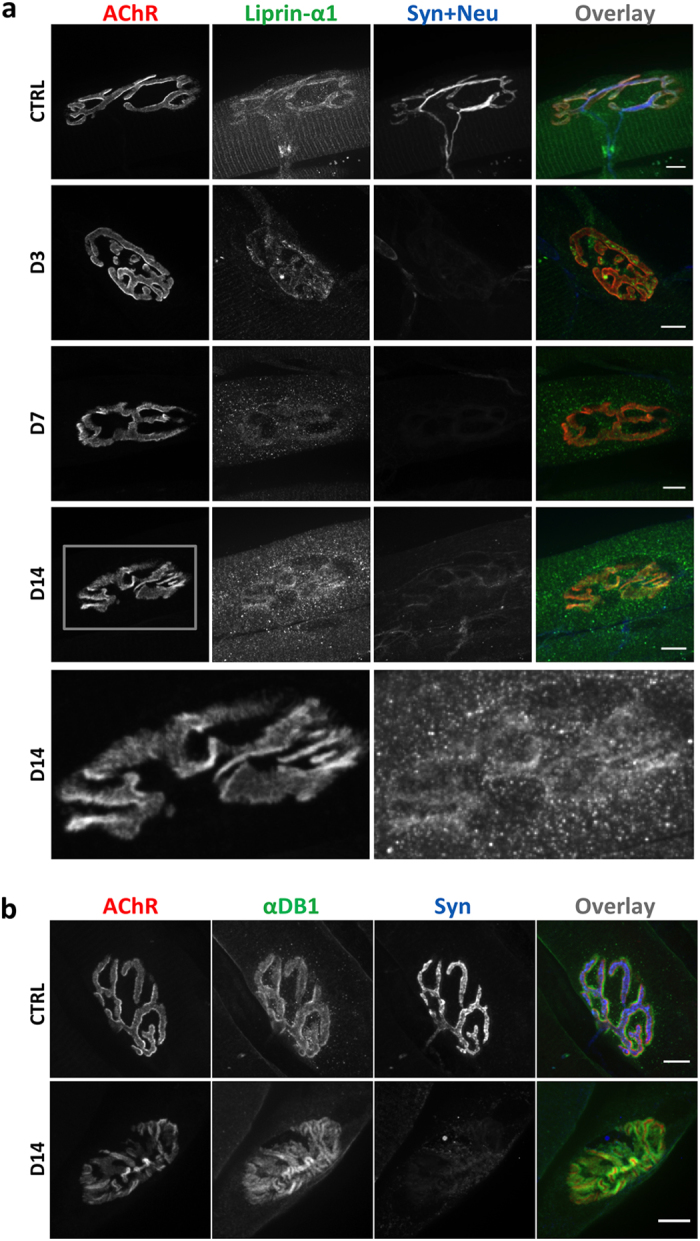
Liprin-α-1 NMJ localization is nerve-dependent. Fibers were isolated from the tibialis anterior muscle from P30 mice on the indicated days after the nerve cut procedure. (**a**) Liprin-α-1 (green) localisation at different days after nerve cut. The bottom panel represents higher magnification of the box in the upper panel (D14). (**b**) α-dystrobrevin-1 immunostaining (green) 14 days after nerve cut. AChR are visualized with BTX in red and anti-synaptophysin/anti-neurofilament immunostaining is shown in blue. All of the images were collected using the same exposure and imaging parameters. Scale bar 10 μm.

When NMJ maturation is accomplished, postsynaptic machinery still undergoes remodelling, including the continuous endocytosis and exocytosis of synaptic components. The instability of postsynaptic machinery can be induced after nerve injury, which leads to the enhanced internalization of AChR. To study potential correlations between increases in NMJ plasticity and the localization of liprin-α-1, we performed a nerve cut procedure that leads to permanent degeneration of the nerve terminal^48, 49^. We carried out immunohistochemical analyses of teased fibers that were isolated from the tibialis anterior muscle after sciatic nerve cut 3, 7, and 14 days after surgery (Fig. 3a). In uninjured, control tissues, liprin-α-1 was present at both pre- and postsynaptic sites and also at sarcomeres. Three days after surgery, the nerve terminal began to withdraw, and AChR became more diffused at the postsynaptic membrane. Interestingly, 7 days after surgery, we observed the mislocalization of liprin-α-1 from the postsynaptic specialization, which became more apparent 7 days later. A similarly striated pattern of liprin-α-1 immunoreactivity along the fibers gradually disappeared following nerve degradation (Fig. 3a). Interestingly, 14 days after denervation αDB1 immunoreactivity was still detected at the postsynaptic machinery, although it appeared slightly more diffuse (Fig. 3b). Although liprin-α-1 interacts with αDB1, its recruitment to the postsynaptic machinery appears to be independent of αDB1, since liprin-α-1 colocalized with AChRs in αDB KO muscles (Supplementary Fig. 2b,c). Collectively, these observations indicate that liprin-α-1 localization in skeletal muscle fibers depends on innervation but not the presence of αDB1 protein.

### Liprin-α-1 is required for AChR cluster formation

**Figure 4:**
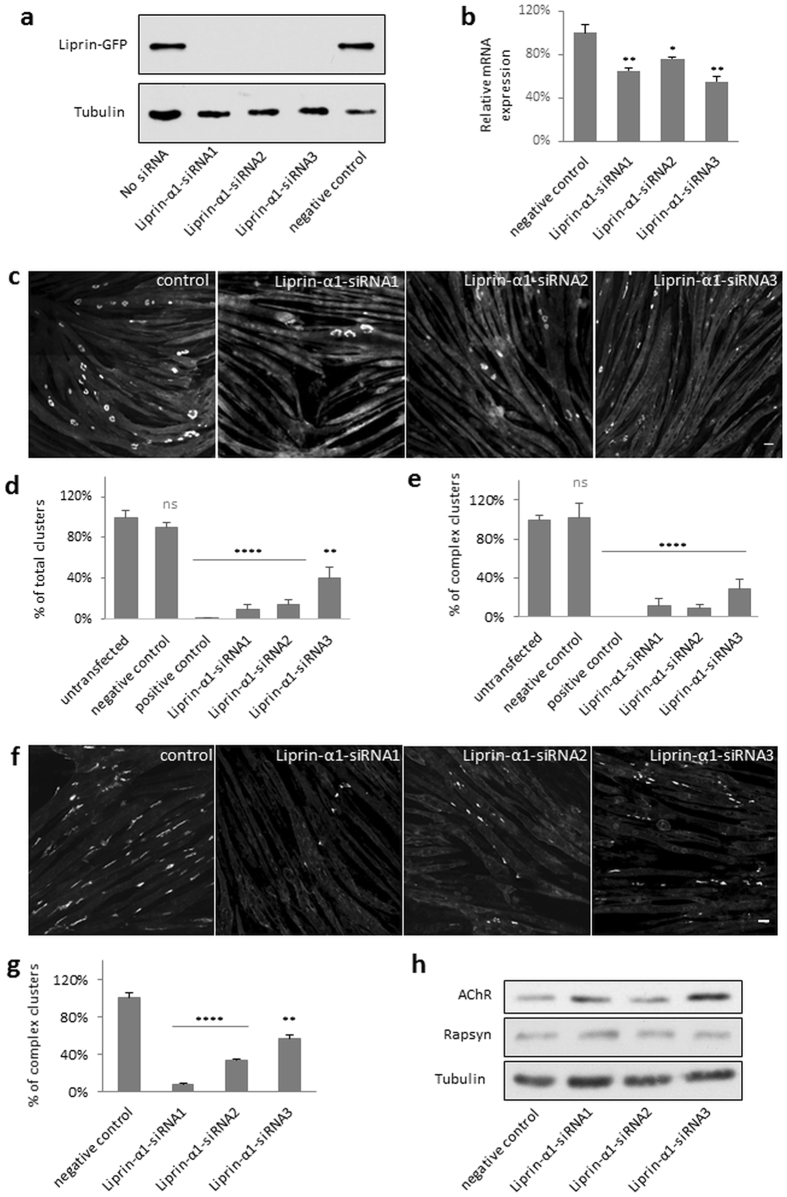
Liprin-α-1 is required for the formation of AChR clusters. (**a**) Validation of liprin-α-1 knockdown efficiency by Western blot. Hek-293 cells were co-transfected with liprin-α-1-GFP and the indicated siRNAs that targeted liprin-α-1 or indicated control siRNAs. Western blot for tubulin was used as a loading control. (**b**) Validation of liprin-α-1 knockdown efficiency by qRT-PCR. C2C12 differentiated myotubes were transfected with indicated siRNAs and 2 days later the total RNA was isolated from cell extract followed by qRT-PCR analysis of liprin-α-1 expression. (**c**) C2C12 myotubes cultured on laminin-coated surfaces were transfected with siRNAs that targeted liprin-α-1 and AChR clusters were visualised with BTX. (**d**) Quantification of the total number of clusters. (**e**) Quantification of topologically complex (perforated) clusters. (**f**,**g**) Liprin-α-1 was essential for the formation of agrin-induced AChR clusters. C2C12 myotubes were cultured on gelatin-coated surfaces, transfected with siRNAs that targeted liprin-α-1, and stimulated with soluble agrin (Z-fragment) to form AChR clusters. (**g**) Quantification of AChR clusters in (**f**). (**h**) Western Blot analysis of AChR and rapsyn expression levels after liprin-α-1 knockdown. Lysates from C2C12 myotubes transfected with liprin-α-1 targeting siRNAs or non-targeting RNAs were probed with antibodies against specified proteins. Levels of tubulin were used as a loading control. Negative control -non-targeting siRNA; positive control - muscle-specific kinase (MuSK) siRNA. Statistical significance was analysed using 2-tailed t-test. *p < 0.05; **p < 0.01; ****p < 0.0001. Error bars represent standard error of the mean (SEM). Scale bar 20 μm.

To determine whether liprin-α-1 plays a functional role in AChR cluster organization, we performed RNA interference (RNAi) knockdown experiments with myotubes that were derived from C2C12 myoblasts using three synthetic small-interfering RNAs (siRNAs) that were validated with regard to knockdown efficiency (Fig. 4a,b). Myotubes that were cultured on laminin-coated surfaces and transfected with non-targeting siRNA (control) formed numerous clusters of AChR that often acquired a complex topology (Fig. 4c), similar to postsynaptic machinery at the NMJ. In contrast, myotubes that were transfected with liprin-α-1-targeting siRNAs presented significantly fewer AChR clusters and fewer topologically complex assemblies (Fig. 4c–e). Liprin knockdown had similar effects on the formation of agrin-induced AChR clusters. The addition of this nerve-derived glycoprotein to myotubes transfected with non-targeting siRNA (control) induced the formation of small AChR clusters scattered at the myotube surface (Fig. 4f). The inhibition of liprin-α-1 expression significantly affected the formation of agrin-induced clusters (Fig. 4f,g).

### Liprin-α-1 regulates AChR clustering by organizing cortical microtubules

**Figure 5:**
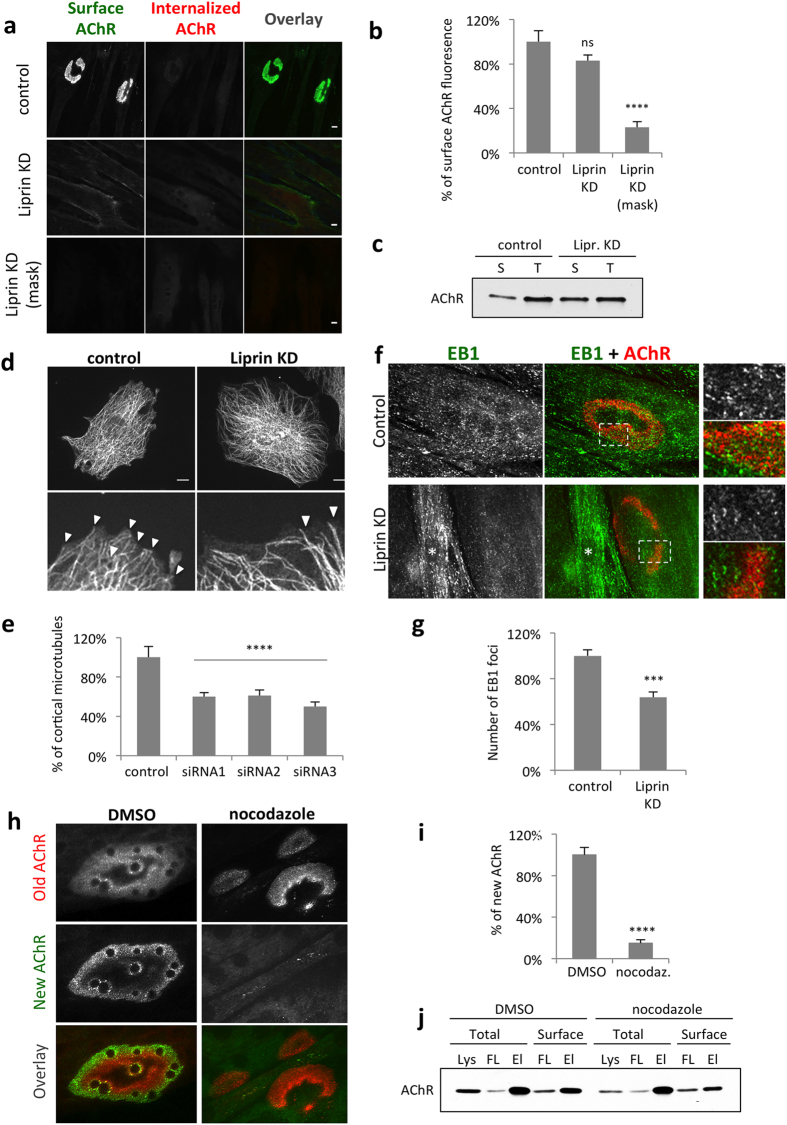
Liprin-α-1 regulates clustering of AChRs by organizing microtubules. (**a**–**c**) C2C12 myotubes depleted of liprin-α-1 have substantial amounts of surface AChR. (**a**) Surface AChR (green) were stained with Alexa-488-BTX and internal AChR were labelled with Alexa-555-BTX. Lower panel shows control experiment for labelling specificity of the Alexa-488-BTX staining shown in the middle panel. Unlabelled BTX was applied to live cells prior to Alexa-488-BTX labelling to mask BTX binding sites on AChR. (**b**) Fluorescence intensity quantification of surface AChR labelling, values normalized to control. (**c**) Analysis of surface AChRs in liprin-α-1-depleted myotubes by AChR precipitation and Western blotting. S-surface and T-total AChR precipitates. (**d**,**e**) Liprin-α-1 knockdown reduces the number of plasma membrane-attached microtubules in myoblasts. (**d**) Cells were transfected with indicated siRNAs and microtubules were visualized with anti-α-tubulin antibody. Lower panel represents higher magnifications of areas in the upper panels. Arrows indicate examples of microtubules in contact with cell surface. (**e**) Quantification of cell surface-attached microtubules per micrometre of the cell edge from 60 cells in three independent experiments. (**f**,**g**) Liprin-α-1 knockdown reduces the number of EB1 foci at the AChR clusters in myotubes. Microtubule tips were visualised with anti-EB1 antibody. Columns at the right represent higher magnifications of boxed areas. Asterisks mark the nucleous of an undifferentiated myoblast. (**f**) Quantification of EB1 foci at from 20 AChR clusters from three independent experiments. (**h**,**i**) Microtubules are dispensable for maintenance of AChR assemblies but are required for the cluster expansion. (**h**) Pre-existing AChR were labelled in live cells with Alexa-555-BTX (Old AChR) and nocodazole or DMSO (control) was added to the cells for 6 h and live cells were labelled again with Alexa-488-BTX (New AChR). (**i**) Fluoresence intensity quantification of surface AChRs. (**j**) Nocodazole treatment does not significantly affect AChR exocytosis. Western blot analysis of surface and total AChR in cell lysates (Lys), flow-through (FL) and elution (EL) fractions from control (DMSO) and nocodazole experiments. Scale bar 10 μm. Statistical significance was analysed using 2-tailed t-test. ****p < 0.0001. Error bars represent SEM.

Defects in AChR cluster formation observed in liprin-α-1-depleted myotubes could be due to the lack of expression of synaptic proteins, impaired cell surface delivery, or impaired assembly of the receptors into postsynaptic structures. Neither levels of AChR receptor subunit α, nor levels of rapsyn, a scaffold protein required for AChR clustering, were reduced in siRNA-transfected myotubes (Fig. 4h). We then checked if the liprin siRNA-mediated reduction of the number of AChR clusters could be due to the defective transport of AChR to the cell surface. For this, we visualized surface AChRs by labelling live cells with Alexa-488-tagged BTX shortly prior to cell fixation. Fixed myotubes were permeabilized and additionally stained with Alexa-555-tagged BTX to label internal AChRs. Liprin-α-1-depleted myotubes failed to assemble AChR clusters observed in control cells, however significant amount of fluorescence was detected at the myotubes surfaces, suggesting that high amounts of dispersed AChRs were still present (Fig. 5a,b). The level of internal AChRs detected with Alexa-555-tagged BTX was unchanged upon liprin-α-1 depletion (Fig. 5a). To eliminate the possibility that Alexa-488 signal (surface AChRs) came from the autofluorescence of the fixed cells we performed a control experiment in which we masked surface receptors by incubation with saturating dose of unlabelled BTX followed by incubation with Alexa-488-tagged BTX. As expected, pre-masking of surface receptors completely blocked the Alexa-488 signal, which proves that our detection method for surface AChRs is specific (Fig. 5a,b). To independently determine the amount of surface AChRs in control and liprin-α-1 knockdown cells, we labeled AChRs with biotin-conjugated BTX either in live cells (to label surface receptors) or in cell lysates (to label total cellular AChR pool) and precipitated both pools of AChR with streptavidin-coated beads. As shown in Fig. 5c, myotubes depleted of liprin-α-1 had significant amounts of AChRs at the plasma membrane.

These experiments suggest that defects in the formation of AChR assemblies upon liprin-α-1 knockdown were not due to the impaired synthesis of AChRs or their insufficient amount at the plasma membrane.

Previous studies by van der Vaart *et al*., suggested that liprin-α-1 plays a role in the anchoring of microtubule ends at the cell cortex in HeLa cells^21^. We therefore examined if liprin-α-1 may have similar function in cultured muscle cells. We first performed microtubule cytoskeleton analysis in myoblasts that were transfected with liprin-α-1 targeting siRNAs or control, non-specific siRNA. Both control and liprin-α-1 depleted myoblasts assembled a dense microtubule network, however, liprin-α-1 knockdown significantly reduced the number of microtubule filaments attached to the cell cortex (Fig. 5d,e). Analysis of microtubule attachment to the plasma membrane in differentiated myotubes is complicated because myotubes make a very dense microtubule network making the distinction of individual filaments and their tips very difficult (see Supplementary Fig. 1). In order to distinguish microtubule tips we stained myotubes for end-binding protein 1 (EB1), a molecular scaffold that is present at the plus-end of microtubules and analysed EB1 density on single plane images of plasma membrane collected with a confocal microscope, as had been done previously^25^. We observed that liprin-α-1 depletion led to significant reduction of EB1 foci at the AChR clusters (Fig. 5f,g).

Interestingly, enrichment of microtubule–plasma membrane contacts specifically at the postsynaptic specialization was shown previously to be crucial for AChR clustering, suggesting that microtubule cytoskeleton plays important role in the receptor aggregation apart from its role in the intracellular trafficking^25^. Moreover, the impaired microtubule attachment at the AChR clusters did not disassemble pre-existing clusters but significantly inhibited formation of new assemblies^25^. These observations prompted us to study if the abrupt disruption of microtubule cytoskeleton with nocodazole treatment has similar effect on AChR organization. For this purpose we labelled pre-existing surface AChRs with Alexa-555-tagged BTX in live cells and applied nocodazole for the next six hours. After this chase period we performed a second labelling of surface AChR with BTX conjugated to Alexa-488, to visualize newly synthesized AChR. In control myotubes (h), newly synthesized AChR were circumferentially incorporated into the postsynaptic assembly surrounding centrally located pre-existing receptors, as published previously^50^. Nocodazole treatment did not affect the organization of the pre-existing AChR clusters, however, it abolished incorporation of the new AChR into the assemblies (Fig. 5h,i). To check if nocodazole treatment abolishes AChR exocytosis we performed precipitation of surface receptors labelled with biotin-conjugated BTX. As shown in Fig. 5j the amounts of surface AChRs were not significantly altered after nocodazole treatment. These results suggest that in cultured myotubes microtubules play an important role in the clustering of AChRs and play a minor role in AChR cell surface delivery.

## Discussion

In vertebrates, liprin-α-1 has been shown to play a role in the organization of synapses in the CNS, but has not been studied at the NMJ. We employed several approaches to characterize the localization of liprin-α-1 at the murine NMJ and its involvement in the formation of AChR clusters in cultured myotubes. Our immunohistochemical analysis and the ectopic expression of GFP-tagged proteins revealed that liprin-α-1 was localized to muscle contractile machinery where it concentrated at Z-discs and co-localized with α-actinin. Additionally, liprin-α-1 was strongly enriched at the NMJ where it was present at both pre- and postsynaptic compartments in different types of muscles. The amount of liprin-α-1 protein at the synapse appeared to be unchanged during development, but its localization was affected by nerve injury. Liprin-α-1 recruitment to the postsynaptic machinery appeared to be independent of interaction with αDB1. This suggests that liprin-α-1 could bind more than one postsynaptic protein. One candidate could be catulin-α-1, which binds to several DGC components^17^, and similarly to liprin-α-1 appeared to localize to the NMJ in the absence of αDB1^12, 51^. Our functional studies revealed that liprin-α-1 is involved in the organization of postsynaptic machinery because it was required for the formation of laminin- and agrin-induced AChR clusters. The defects in the formation of AChR clusters observed with liprin-α-1 knockdown were more severe than those observed in αDB knockout cells^12, 52^. This suggests that liprin-a1 has additional functions at the NMJ, independent from αDB1. Our and previous studies^19, 21^ demonstrated that it is involved in the organization of plasma membrane-attached microtubules. Basu *et al*., have shown that LL5β-dependent attachment of microtubule tips to the postsynaptic membrane plays a crucial role in the AChR clustering^25^. Importantly, LL5β knockdown appeared to have no effect on the pre-existing AChR clusters, but inhibited aggregation of the newly synthesized AChR^24, 25^. In agreement with these observations, our experiments on acute disruption of microtubule cytoskeleton with nocodazole showed that the microtubules are dispensable for the maintenance of the pre-existing AChR clusters but are required for the cluster expansion in C2C12 myotubes (Fig. 5h–j). Microtubules have an important function in intracellular vesicular trafficking, and an obvious possibility would be that microtubule disassembly blocks biosynthetic transport to the plasma membrane. Connolly J.A., however, demonstrated that disruption of microtubules with nocodazole in cultured chick myotubes had a minor effect on the AChR transport to the cell surface and newly synthesized AChR were efficiently exocytosed^53^. This is in agreement with our observation that disassembly of the microtubule cytoskeleton with nocodazole does not substantially alter AChR delivery to the plasma membrane (Fig. 5j). Similarly, liprin-α-1 depleted myotubes appeared to have significant amount of AChR at the cell surface that failed to aggregate (Fig. 5a–c). Also LL5β knockdown, which blocks clustering of AChR on myotubes surfaces^24^ was proposed to reduce the density specifically of AChR-associated cortical microtubules^25^. These observations argue that microtubules attached at the postsynaptic machinery could have important functions in the clustering of the postsynaptic components apart from their role in exocytosis. It is believed that microtubules are utilized for vesicular transport over longer distances and actin filaments play a more important role in short-distance delivery^54, 55^. At the neuromuscular junctions, as well as at the laminin-cultured myotubes synaptic genes are expressed predominantly from specialized postsynaptic nuclei that are clustered directly underneath the postsynaptic membrane^50^. Such compartmentalization together with local synthesis leads to the enrichment of synaptic mRNAs and proteins at the synapse, thus reducing the need for long-distance transport. Therefore, vesicular transport to the muscle postsynaptic specialization could rely more on the actin cytoskeleton. Microtubules could have either mechanical or signalling role, which may influence clustering of AChR, but the underlying mechanism is not clear. It could be possible that local exocytosis directed specifically to the AChR cluster^25^ could be crucial for AChR incorporation into the assembly. Recent studies utilizing quantum dots, however, provided evidence supporting the diffusion-trapping hypothesis for incorporation of AChR into the postsynaptic membrane^56^. If this is the case, one would expect circumferential localization of newly incorporated receptors as shown in Fig. 5 and reported previously for AChR clusters formed by laminin-cultured myotubes and postsynaptic machinery *in vivo* ^50^.

The role of liprin-α-1 at the postsynaptic membrane may not be restricted to the regulation of cortical microtubules. We have recently demonstrated that liprin-α-1 binds to αDB1 and Lyssand *et al*. showed that it also interacts with catulin-α-1 in HEK-293 cells^17^. These two cytoplasmic DGC-associated proteins play an important role in the organization of the postsynaptic apparatus^12, 13^. Liprin-α-1 may also function as a scaffold, recruiting signalling proteins such as: LAR transmembrane protein-tyrosine phosphatase (which controls the development of invertebrate NMJs)^57^, MAGUK (a kinase that is involved in the muscle-specific kinase [MuSK] signaling pathway at the vertebrate NMJ)^58^, and CamKII (which is critical for AChR recycling)^59^. de Curtis and colleagues proposed that the liprin-α-1 and its binding partners function as a hub for the recruitment of additional effector proteins that form dynamic supramolecular assemblies at specific membrane locations, such as presynaptic nerve terminals, the leading edge of migrating cells or invasive protrusions called podosomes^18, 19^. Thus, additional studies should be conducted to shed more light in the function of this important synaptic protein.

## Methods

### Cell culture

C2C12 cells were purchased from the American Type Culture Collection (CRL1772) and cultured for up to five passages in Dulbecco’s Modified Eagle Medium (DMEM) supplemented with 20% fetal bovine serum (FBS), 4.5 g/L glucose, L-glutamine, penicillin, streptomycin, and fungizone on 0.2% gelatin-coated plastic dishes. Prior to the experiment, the cells were trypsinized and plated on Permanox slides in eight-well Flexiperm chambers (catalog no. 177445, Sigma-Aldrich). The slides were coated with laminin 111 (catalog no. 23017-015, Invitrogen) in DMEM. To induce cell fusion, the media were replaced with DMEM that contained 2% horse serum, 4.5 g/L glucose, L-glutamine, penicillin, streptomycin, and fungizone.

For the agrin experiments, slides were coated with 0.2% gelatin and myotubes were stimulated with 10 nM agrin (catalog no. 550-AG-100/CF, Biokom). Hek293 cells were from the American Type Culture Collection (CRL-1573) and maintained in DMEM with 10% FBS, 4.5 g/L glucose, L-glutamine, penicillin, streptomycin, and fungizone.

Primary myoblast cultures were performed as described previously^60, 61^. Briefly, tibialis anterior and extensor digitorum longus muscles were digested with 0.2% collagenase in DMEM for 1, 5 h. Next, muscles were transferred to DMEM containing 10% horse serum, 0.5% chicken embryo extract and penicillin and streptomycin and single fibers were isolated from dissociated muscles. Fibers were stripped using syringe with 21-gauge needle. Cells were filtered through 70 µm nylon cell strainer and plated on Flexiperm chamber slides covered with laminin and agrin^28^. Myoblasts were cultured in DMEM containing 20% FBS, 10% horse serum, 0.5% chicken embryo extract, penicillin and streptomycin. After 4 days of culture myoblasts were transfected with liprin-α1-GFP plasmid using Lipofectamine 2000 (catalog no. 11668-030, ThermoFisher) according to manufacturer’s instructions. Six days after transfection cells were fixed in 4% PFA and labeled with AlexaFluor-555-conjugated BTX.

### siRNA transfection

Differentiated C2C12 myotubes were transfected with 20 nM siRNAs (Qiagen) that targeted liprin-α-1 (catalog no. and targeted sequences: siRNA1 [SI04927860, CTGCTTGACGGAAACCATGAA], siRNA2 [SI04927867, CACGTCTGTGCATGACCTCAA], siRNA3 [SI04927874, ATCCTGTCGATTGGCCTTAAA]) using Lipofectamine RNAiMAX (catalog no. 13778075, Life Technologies) at 48–72 h after fusion according to the manufacturer’s instructions. The cells were fixed 2–3 days later and examined by confocal microscopy. Transfections of HEK293 cells were performed with Lipofectamine 2000 Transfection Reagent.

### Cell staining

The cells were fixed in 4% paraformaldehyde (PFA) and stained using AlexaFluor-555-bugarotoxin (BTX) (catalog no. B35451, Life Technologies) and Alexa-488-taged BTX (catalog no. B13422, Life Technologies). For labelling of AChRs in live cells BTX conjugates were added to the culture media for 5–7 min; internal AChR staining was performed after cell membrane permabilization with 0.5% Triton X-100 for 1 h. For masking of BTX binding sites on surface AChRs unlabelled BTX (catalog no. B1601, Life Technologies) was added to the culture media for 20 minutes. For microtubule disruption 10 µg/ml nocodazole (catalog no. M1404, Sigma) or DMSO (control) was added to the media for 6 hours. Microtubules were visualised with anti-tubulin (catalog no. ab18251, Abcam) and EB1 with anti-EB1 (catalog no. 610534, BD Biosciences). F-actin was stained with Acti-stain-670 (catalog no. PHDN1, Cytoskeleton).

### Microscopy and image analysis

Microscopic analysis was performed at the Confocal Microscopy Facility, Nencki Institute, using a Zeiss Spinning Disc confocal microscope or Leica TCS SP8 scanning confocal microscope that were equipped with diode or white light lasers, respectively. Images were collected using ZEN software (ZEISS International) and analysed using ImageJ/Fiji software. Enrichment of liprin-α-1 at the NMJ postsynaptic machinery was analysed using RGB profiler plugin (https://imagej.nih.gov/ij/plugins/rgb-profiler.html) at the ImageJ/Fiji software. Quantitative image analysis was performed using ZEN Lite software (ZEISS International). For the quantification of surface AChRs, mean fluorescence counts from whole images was collected. Values obtained for liprin-α-1 knockdown and masking experiments were calculated as percentages of the mean fluorescence from the negative control.

### Western blotting and AChR precipitation

The cells were lysed in lysis buffer (50 mM Tris-HCl, 150 mM NaCl, 1% Nonidet-P40, 0.5% sodium dodecyl sulfate [SDS], 10% glycerol, 1 mM dithiothreitol [DTT], 1 mM NaF, and ethylenediaminetetraacetic acid-free mini protease inhibitor cocktail [catalog no. 11873580001, Roche], pH 8.0). The cells were scraped off the dish, incubated briefly on ice, passed three times through a 25-gauge needle, incubated on ice for 15 min; and centrifuged at 20,000 × *g* for 30 min, at 4 °C. For Western blot, the supernatant samples were mixed with sample buffer and boiled for 5 min. The proteins were then resolved by SDS-polyacrylamide gel electrophoresis and transferred to nitrocellulose membranes (catalog no. 66485, Pall Corporation) using Trans Blot Turbo (catalog no. 1704270, Bio-Rad). Membranes were blocked with 5% milk in TBST (20 mM Tris-HCl, 150 mM NaCl [pH 7.6], and 0.1% Tween20) for 1 h at room temperature and probed with primary antibodies that were diluted in 5% milk in TBST at 4 °C overnight. After washing with TBST buffer, the membranes were incubated with appropriate secondary antibodies conjugated to horseradish peroxidase. For protein detection, we used Clarity chemiluminescent substrate (catalog no. 1705060, Bio-Rad). The primary antibodies were anti-GFP (catalog no. ab32146, Abcam), anti-AChR-α1 (catalog no. 10613-1-AP, Proteintech), anti-rapsyn (catalog no. ab156002, Abcam) and anti-tubulin (catalog no. ab18251, Abcam).

For precipitation of surface AChR^62^, BTX-biotin (catalog no. B1196, Invitrogen) was added to the media for 5 minutes at 37 °C, washed with PBS, followed by cell lysis. For precipitation of total AChR, cells were lysed and incubated with BTX-biotin for 30 minutes at 4 °C. Lysates were incubated with NeutrAvidin beads (catalog no. 29200, Thermo Scientific) overnight in 4 °C. Beads were collected by centrifugation (2500x g), washed three times with wash buffer (100 mM Hepes pH 7.4, 150 mM NaCl, 1 mM PMSF, 0.5% NP-40), resuspended in 2x sample buffer and boiled. Rat polyclonal antibody (catalog no. MRT-609R, BioLegend) was used to detect precipitated AChR.

### Sciatic nerve cut and plasmid electroporation

P30 mice were anesthetized with a ketamine (150 mg/kg)/xylazine (10 mg/kg) cocktail, and the surgery area was shaved and disinfected with ethanol. A 1 cm incision was made in the longitudinal direction proximal to the knee and a 5 mm fragment of the sciatic nerve was removed with sterile scissors. The skin was sutured and Lidocaine was applied to the wound, and analgesics were administered.

DNA electroporation was performed under general anesthesia (see above), and 25 μg DNA (1 μg/μl) was injected into the tibialis anterior muscle using a Hamilton syringe. The muscle was electroporated with 10 pulses of electrical current (180 V/cm) of 20 ms each at 1 s intervals using an ECM-830 electroporator (catalog no. 45-0052INT, BTX Harvard Apparatus). Lidocaine was applied to the wound and analgesics were administered. The animals were sacrificed 14 days after surgery.

αDB KO mice^63^ were a kind gift from Joshua R. Sanes (Harvard University, USA). C57BL6 mice were obtained from Jackson Laboratories and maintained at the Nencki Institute of Experimental Biology in the animal facility equipped with a selected pathogen free (SPF) barrier (standard). For the animal experiments, we obtained approval from the First Warsaw Local Ethics Committee for Animal Experimentation, and all of the experiments were conducted in accordance with all applicable laws and regulations. The health of the animals was monitored by veterinarian on-staff.

### Muscle tissue preparation

The mice were sacrificed by a lethal injection of pentobarbital (200 mg/kg). Mouse embryos were extracted at E16. The tibialis anterior, triangularis sterni, soleus, and gastrocnemius muscles were excised and fixed in 4% PFA or cold methanol. Muscles were stripped of connective tissues and either used for the preparation of teased fibers or stained in a whole-mount procedure. Nonspecific staining was blocked with 2% bovine serum albumin and 2% goat serum in PBS supplemented with 0.5% Triton-X100 before overnight incubation with primary antibodies. The muscles were stained using fluorescently labeled BTX (catalog no. B35451, Life Technologies) and primary antibodies against anti-liprin-α-1 (catalog no. 115098, GeneTex), anti-α-actinin (catalog no. sc-17829, Santa Cruz Biotechnology), anti-ryr (catalog no. ab2868, Abcam), anti-myomesin (catalog no. ab111433, Abcam), anti-α-dystrobrevin-1 (catalog no. 610766, BD Transduction Laboratories), anti-neurofilament (catalog no. 2H3, Developmental Studies Hybridoma Bank), and anti-synaptophysin (catalog no. 101 004, Synaptic Systems). For cryosectioning tibialis anterior muscles were flash-frozen in isopentane cooled with liquid nitrogen and sectioned at 10–20 µm in a cryostat. Cryosections were fixed and stained as described above.

### DNA constructs, RNA isolation and quantitative PCR analysis

The liprin-α-1-GFP (human) plasmid was a kind gift from Ivan de Curtis (San Raffaele Scientific Institute, Milano, Italy)^20^. The liprin-α-1-GFP (mouse) plasmid was constructed by amplification of the liprin-α-1 coding sequence from pLiprin plasmid (catalog no. IRCKp5014O0117Q, Source BioScience) using GATCGCTAGCATGATGTGCGAGGTGATGCCGACCATTAGT and CATTGGTACCAGCAGGAGTAAGTCCTGACTGTGGCAGAGTC primers and subcloned into the pEGFP-N2 vector.

RNA was isolated with Trisure Reagent (catalog no. BIO-38032, Bioline) according to manufacturer’s instructions. cDNA was generated from 1 µg of templates using the High Capacity cDNA Reverse Transcription Kit (catalog no. 4374966, Applied Biosystems). RT-qPCR was performed in triplicates for RNAs obtained from two independent experiments using TaqMan Fast Universal PCR Master Mix (catalog no. 4352042, Applied Biosystems) with predesigned liprin-α1 TaqMan probe (catalog no. 4448892, ThermoFisher) on Step One Plus instrument (Applied Biosystems). Results were analyzed with Step One software v2.3 using the standard curve method and standardized to 18 S rRNA (catalog no. 4333760, ThermoFisher).

### Data Availability

The datasets generated during and/or analysed during the current study are available from the corresponding author on reasonable request.

## Electronic supplementary material

Supplementary Information
