# Supplementary material for: Liprin-α-1 is a novel component of the murine neuromuscular junction and is involved in the organization of the postsynaptic machinery
Source: Sci Rep. 2017 Aug 22;7:9116. doi: 10.1038/s41598-017-09590-7 (PMC5567263; doi:10.1038/s41598-017-09590-7)
Supplement: Supplementary file 1 — Supplementary Information [file 41598_2017_9590_MOESM1_ESM.pdf]

Liprin-α-1 is a novel component of the murine neuromuscular junction and is involved in the organization of the postsynaptic machinery

Krzysztof M. Bernadzki[a], Marta Gawor[a], Marcin Pęziński[a], Paula Mazurek[a], Paweł Niewiadomski[a], Maria J. Rędowicz[b], and Tomasz J. Prószyński[a]*

[a]Laboratory of Synaptogenesis and [b]Laboratory of Molecular Basis of Cell Motility, Nencki Institute of Experimental Biology, Polish Academy of Sciences, 3 Pasteura Street, Warsaw 02-093, Poland

*Corresponding Author:

Tomasz J. Prószyński

Laboratory of Synaptogenesis

Nencki Institute of Experimental Biology

3 Pasteura Street

02-093 Warsaw, Poland

t.proszynski@nencki.gov.pl

Keywords:

Liprin, AChR, neuromuscular, NMJ, postsynaptic machinery, synapse, microtubules

Figure legends

Fig. S1. Dense microtubule structures in C2C12 myotubes. C2C12 myotubes grown on laminin were stained for AChR (BTX, red) and tubulin (green) and imaged with scanning confocal microscope. Image of a single focal plane is shown; scale bar 10 µm.

Fig. S2. Liprin-α-1 localisation at the postsynaptic machinery in C2C12 myotubes and αDB KO cells. (a) Liprin-α-1-GFP (green) is associated with AChR clusters (red) in laminin-cultured myotubes. GFP signal is detected underneath AChRs and at the cortex-like domain surrounding F-actin rich domain (blue) of synaptic podosomes [1-3]. Lower two panels represent magnification of areas shown in the upper panel. (b) Liprin-α-1-GFP (green) is associated with AChR clusters (red) in myotubes derived from wild type and αDB KO myotubes. (c) Imunohistochemical analysis of liprin-α-1 localisation (red) at the NMJ (AChRs visualised with BTX, green) in cryostat cross-sections of tibialis muscle from wild type and αDB KO mice. Scale bar 10 µm.

References

1 Bernadzki, K. M., Rojek, K. O. & Proszynski, T. J. Podosomes in muscle cells and their role in the remodeling of neuromuscular postsynaptic machinery. *Eur J Cell Biol* **93**, 478-485, doi:10.1016/j.ejcb.2014.06.002 (2014).

2 Proszynski, T. J., Gingras, J., Valdez, G., Krzewski, K. & Sanes, J. R. Podosomes are present in a postsynaptic apparatus and participate in its maturation. *Proc Natl Acad Sci U S A* **106**, 18373-18378, doi:10.1073/pnas.0910391106 (2009).

3 Proszynski, T. J. & Sanes, J. R. Amotl2 interacts with LL5beta, localizes to podosomes and regulates postsynaptic differentiation in muscle. *J Cell Sci* **126**, 2225-2235, doi:10.1242/jcs.121327 (2013).

Fig. S1

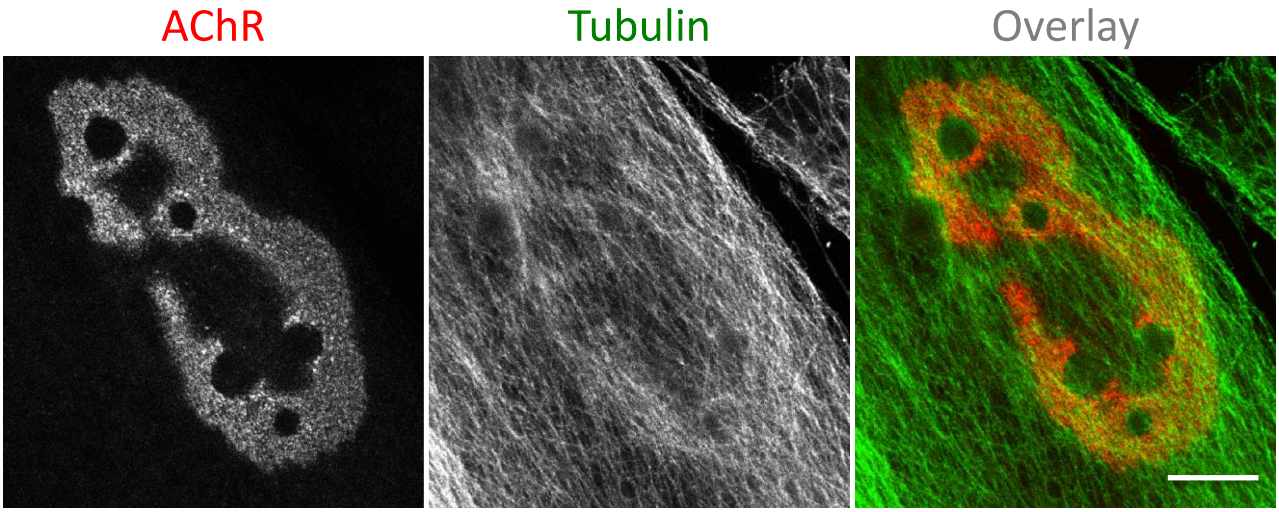

Fig. S2

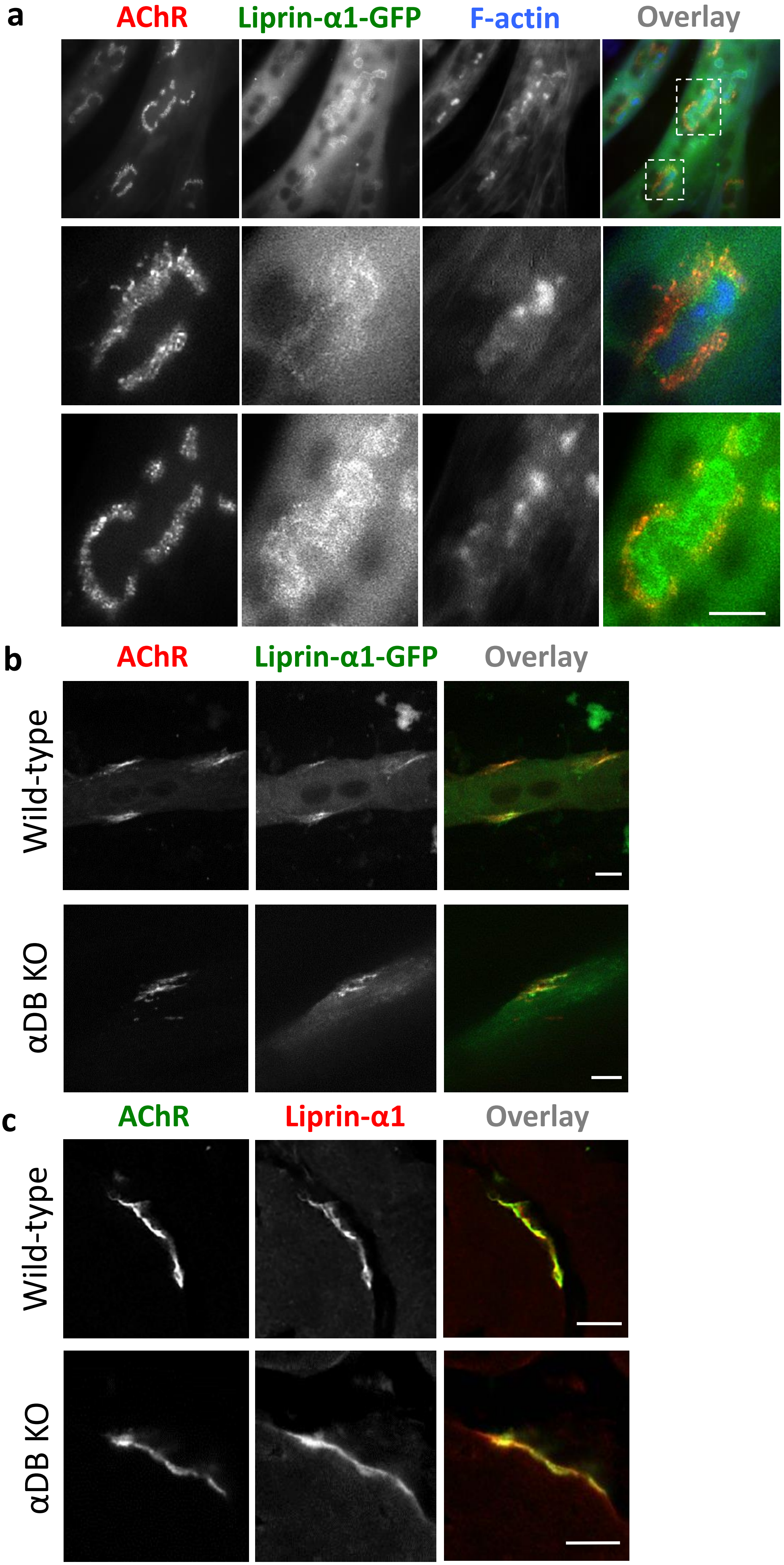

a
AChR
Liprin-α1-GFP
F-actin
Overlay
b
AChR
Liprin-α1-GFP
Overlay
Wild-type
αDB KO
c
AChR
Liprin-α1
Overlay
Wild-type
αDB KO
